# Accurate surgery time prediction (ASTP) strategy based on artificial intelligence techniques

**DOI:** 10.1038/s41598-026-55198-1

**Published:** 2026-06-12

**Authors:** Rana Mohamed El-Balka, Asmaa H. Rabie, Ahmed I. Saleh, Noha Sakr

**Affiliations:** 1 Computer and Control Systems Engineering Department, Faculty of Engineering, Mansoura University, Mansoura, Egypt; 2 Computer Science Department, Arab East Colleges, Arab, Saudi Arabia

**Keywords:** Histogram Gradient Boosting Regression, Long Short Term Memory, Machine learning, Operating room, Prediction Time, SHapley Additive exPlanations, Computational biology and bioinformatics, Engineering, Health care, Mathematics and computing, Medical research

## Abstract

Accurate timing prediction of surgery is essential for efficient operating room scheduling and ensuring patient care. This study proposes a two-layered Accurate Surgical Time Prediction (ASTP) framework. The first layer combines feature importance and advanced machine learning models to estimate surgical time. After preprocessing, two complementary interpretable AI methods were used: Long Short Term Memory with SHapley Additive exPlanations (SHAP) values and Random Forest permutation importance, to determine the importance of features. In the second layer, subsets of features (TOP-K) were progressively evaluated using HGBR and compared with multiple models: artificial neural networks (ANNs) and recurrent models (Long Short Term Memory, Gated Recurrent Unit, and hybrid Long Short-Term Memory + Gated Recurrent Unit). The proposed framework was evaluated on two datasets: a real-world dataset from an operating room at Nile Hospital and a public dataset from the Medical Informatics Operating Room Vitals and Events Repository (MOVER). In the Nile Hospital dataset, the results show that the Histogram Gradient Boosting Regression (HGBR) approach achieves the best balance, with a Mean Absolute Error of 8.89 min, Root Mean Square Error of 19.5 min, and R-squared of 0.26 using only four features, outperforming other methods. On the MOVER dataset, HGBR also demonstrated the strongest overall predictive behavior, achieving its best numerical result at TOP-12 and best subset at TOP-7, which preserved near-optimal performance with reduced input complexity. The proposed ASTP framework provides an interpretable and resource-efficient solution for surgical time prediction, supporting more intelligent operating room scheduling and facilitating future integration into hospital decision-making.

## Introduction

Healthcare systems are one of the most important service sectors in every country. Hospitals play an important role in health systems, and operating rooms (ORs) are the most expensive services, requiring multiple precious resources such as surgeons, personnel, and equipment. Optimizing operating room utilization is a pivotal aspect of hospital management strategy. It is essential to strategically organize surgical schedules, taking into account the availability of hospital beds and medical staff, while prioritizing patient safety. A fundamental and critical step in organizing an operating room is assessing and predicting the duration of the surgical procedure^1,2^. However, predicting the duration of surgery is difficult due to many uncertain factors, such as patient condition, changes in the surgical plan, problems with surgical instruments, and unexpected bleeding^3,4^. In several hospitals, operating rooms are managed depend on common methods for predicting surgical duration, such as applying average durations for specific procedures or based on the anesthesiologist’s subjective opinion^5^.

Inaccurate estimates of surgical duration increase hospital operating expenses, lengthen healthcare staff working hours, and lead to dissatisfaction, burnout, and an increased risk of medical errors^6^. Human error is often caused by individual surgeon factors, as well as work system factors, meaning that human error is the major contributing factor to “non-repeat events”. Human error includes surgeon distraction, lack of awareness of the surgical team about the status of potential errors, and poor communication between team members. In addition, institutional factors and working conditions, including increased workload and physician stress, can create a work climate that is unfavorable for achieving the standards required to maintain patient safety and teamwork effectiveness^7^. Addition, it leads to longer patient waiting times, fasting, and increased patient and family dissatisfaction. This highlights the importance of developing a more accurate and reliable method for predicting surgical duration. The use of machine learning algorithms in operating room management has been proposed to significantly enhance case duration prediction by varying input factors or feature^8^.

The AI and machine learning approach to operating room management began with the discovery that healthcare data, from patient demographics to surgical records, anesthesia regimens, and recovery room dynamics, holds untapped potential. In 2015, machine learning research in medicine expanded significantly, moving from theory to practical applications. The healthcare sector is benefiting from a deeper understanding of machine learning and greater processing power to tackle complex problems^9^. In the era of data-driven healthcare, machine learning (ML) has become a cornerstone of operating room tasks, predicting surgical duration, improving scheduling, and maximizing resource utilization^10,11^. Several studies have used machine learning algorithms to predict the times of different surgical procedures.

This paper presents a new, efficient framework for operating-room decision support called Accurate Surgery Time Prediction (ASTP), which consists of two layers. In first layer, preprocessing comprises two stages: (i) mixed scaling consisting of one-hot encoding for categorical features, standardization StandardScaler for continuous features, and min–max scaling where appropriate; and (ii) feature ranking, where we determine the feature order using permutation ΔMAE from a tuned Random Forest and LSTM-based SHAP. In second layer, prediction uses a Histogram Gradient Boosting Regression (HGBR) as the final model. Important considerations include rapid re-prediction for last-minute changes, robustness to outliers (winsorization + log1p on train only), and providing transparent drivers of predictions to avoid surgeon/nurse over-allocation and support timely patient care. So unlike existing methods that focus only on deep or tree-based models, the proposed ASTP strategy uniquely integrates interpretable feature-ranking (SHAP + ΔMAE) with resource-efficient regression (HGBR) to generate explainable surgical duration estimates using minimal data.

In addition to improving prediction accuracy, the proposed ASTP framework is designed to address several objectives. Specifically, it aims to: (1) provide accurate prediction of surgical time, (2) identify and interpret the most influential predictors, (3) minimize the input space by selecting the best K feature, and (4) enhance practicality through computationally efficient prediction.

The contributions of this paper are summarized as follows:

- A unified two-layer framework for Accurate Surgical Time Prediction (ASTP) is proposed for estimating surgical duration. This framework consists of a preprocessing and feature ranking layer and a final prediction layer based on the HGBR algorithm.
- A dual feature ranking strategy based on interpretability is developed. This strategy combines LSTM-based SHAP analysis with the permutation importance in a random forest algorithm, enabling precise identification of the most useful features during the perioperative.
- A progressive TOP-K evaluation scheme is presented to assess combined feature subsets and identify efficient predictive configurations with reduced input dimensions.
- Validating the proposed framework on two datasets: the Nile Hospital dataset and the MOVER public dataset.

This paper is organized as follows: Sect. 2 presents the previous efforts in operating room time prediction. Section 3 focuses on the proposed Accurate Surgery Time Prediction strategy. Section 4 the experimental results. The conclusions are discussed in Sect. 5.

## Literature review

Surgical and healthcare technologies are constantly evolving rapidly. However, this evolution poses new challenges, such as long surgical waiting lists and overcrowded operating rooms, which lead to a mismatch between patient needs and available resources. In this section, we discuss surgical duration prediction strategies, along with modern machine learning methods for surgical duration prediction and operating room planning. These methods aim to reduce waiting times, optimize resource allocation, manage surgical delays, enhance efficiency, balance staff workloads, reduce operational costs, and accommodate each patient’s individual priorities. In^12^ suggested develop, implement, and test a semi-automated machine learning technique that uses the present phase and tools to forecast the Remain Surgery Duration (RSD) in laparoscopic cholecystectomy procedures. We use the annotated instrument and stage information from Cholec80, a freely available dataset. This approach is based on a random forest regression model that takes into account two data sources: the surgical stage and the type of instruments used at each time point throughout the procedure. Kwong, Michel, et al.^13^ used electronic health record (EHR) data from three academic centers of higher learning to train models to predict “case duration,” defined as the time between a patient’s entry into the operating room and their exit from the operating room. Model performance was evaluated based on its predictive accuracy and residual analysis, and then compared to the “scheduled duration,” defined as the case duration estimated preoperatively by primary surgeons. Predictive models included simple linear regression, ridge regression, Lasso regression, support vector regression (SVR), random forest, gradient boosting machine, XGBoost, and artificial neural network (ANN).

Riahi, Vahid, et al. proposed developing efficient machine learning (ML) methods to provide more accurate predictions of surgical duration, especially in the absence of surgeon estimation. Individual patient characteristics and prior surgery information from medical records were used to train predictive models. A wide range of algorithms, such as extreme gradient boosting (XGBoost) and random forests (RF), were tested to predict surgical duration^14^.Gabriel, Rodney Allanigue, et al.^15^ compared machine learning models that utilized surgical and patient characteristics in our institutional approach, which utilized historical averages and surgeon-specific adjustments as needed. Multivariate linear regression, random forest, bagging, and XGBoost (extreme gradient boosting) were applied, and the mean R2, root mean square error (RMSE), explained variance, and mean absolute error (MAE) were calculated using k-fold cross-validation. We then used the Shapley’s Additional Explanations (SHAP) model to determine the significance of the features.

Jiao, York, et al.^16^ proposed an artificial neural network and compared with a Bayesian approach and the duration of scheduled surgery. The continuous ordered likelihood score (CRPS) was used as a measure of temporal error to assess the accuracy of the model. To evaluate clinical performance, the accuracy of each approach in identifying cases that exceeded 3:00 p.m. (typically the end of the scheduled shift) was evaluated, identifying opportunities to avoid overtime costs. In^17^ Linear regression, categorical boosting, and feedforward neural networks were trained with embedding created from bidirectional encoder representations from transformers or a transformer model pre-trained on clinical literature. Langenberger, Benedikt, et al.^18^ proposed extreme gradient boosting (XGBoost) and multivariate linear regression were used for prediction. To determine whether machine learning can predict hospital length of stay (DOS) in HA/KA patients using available preoperative retrospective data with reasonable performance, to compare whether machine learning can outperform multivariate regression in predictive performance, and to identify the most important predictors of DOS in both a multi-hospital and single-hospital context. In^19^, the authors proposed improving prediction accuracy beyond traditional estimation methods by developing random forest models specifically designed for specific surgical departments. Using a comprehensive dataset, several machine learning algorithms were applied, including RandomForest, XGBoost, Linear Regression, LightGBM, and CatBoost.

Levin et al.^26^ proposed a gradient-boosted decision-tree machine learning model for the duration of a total shoulder replacement procedure using surgeon, patient, and shoulder-specific variables, highlighting the utility of machine learning in operating room prediction tasks. Ramamurthi et al.^27^ applied large language models to predict the length of surgical cases and reported that finely tuned large language models can achieve similar or better performance than institutional scheduling methods when rich unstructured textual data are available Table 1.

**Table 1:** Comparison of the latest approaches for surgical time prediction.

References	year	Techniques	Limitations	Advantages	Performance	Dataset
Kostopoulos, Spiros, et al.^12^	2025	Semi-automated approach using random forest regression with a dual model strategy (long vs. short surgeries) based on elapsed time	depends on manual annotations	Real-time usability with structured inputs	MAE = 5.89 min MAE = 4.61 min at 20 min before surrey end	Cholec80
Kwong, Michel, et al.^13^	2025	linear regression, Ridge, Lasso, SVR, Random Forest, GBM, XGBoost, ANN	Focused only on general elective surgery	Multi-center data	ANN (MAE ≈ 31.8 min, RMSE ≈ 49.7 min)	Three tertiary academic centers
Riahi, Vahid, et al.^14^	2023	XGBoost, Random Forest	Focus on elective surgeries	Large dataset	XGBoost model reduced the total absolute error by 6854 min	Australian hospitals
Gabriel, Rodney Allanigue, et al.^15^	2023	Ensemble learning models	Focused only on spine surgery	Improving prediction in specialized surgeries	linear regression (explained variance score = 0.345, an R2 = 0.34, RMSE = 162.84 min, MAE = 127.22 min) XGBoost regressor (explained variance score = 0.778, R2 = 0.770, RMSE = 92.95 min, MAE = 44.31 min)	dataset from specialized clinical center
Jiao, York, et al.^16^	2022	ML models compared with Bayesian models	Complexity	Real-time adaptability	ANN (mean = 13.8, standard deviation = 35.4 min)	Intraoperative data: pre-operative information + real-time vital signs and medication use
Ramamurthi, Adhitya, et al.^17^	2025	ML models using Bi-encoder/Clinical BERT+ categorical boosting, compared to linear regression	single-center dataset	Leverages rich clinical text data	Categorical regression model using bidirectional encoder representations (MARE, 46.4 min)	single quaternary care hospital
Langenberger, Benedikt, et al.^18^	2025	XGBoost	Focuses only on elective orthopedic surgeries (not applicable to all types)	Model validated on independent test sets	MAE ≈ 12.1 min	Large Electronic health records dataset from a German hospital
Park, Jung-Bin, et al.^19^	2025	Random Forest (general vs. department-specific models)	Requires large volumes of cases per partition for reliable forecasting.	interpretable with SHAP analysis	MAE = 16.3 min, RMSE = 31.2 min, R² = 0.92 in Random Forest (department-specific) MAE ≈ 34.1 min, RMSE ≈ 57.5 min, R² ≈ 0.72 Random Forest (general)	Electronic health records dataset from a Korean tertiary hospital

## The proposed accurate surgery time prediction (ASTP) strategy

**Fig. 1:**
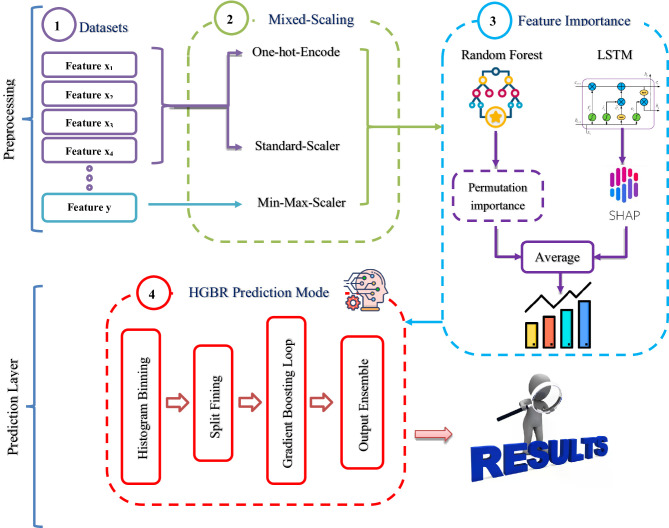
roposed two-layer accurate surgery time prediction (ASTP) framework.

Our strategy consists of two layers as illustrated in Fig. 1. The preprocessing layer performs Mixed-Scaling, computes feature importance using LSTM and Random Forest, and Feature ranking, which orders the features and selects the most informative subset. The prediction layer applies HGBR to the ranked features, yielding the final time-to-surgery prediction.

### Preprocessing layer in ASTP

In this layer, the data is processed using One Hot encoding, Mixed-Scaling, and determining the importance and ranking of features.

#### One hot encoding

In tabular data modeling, each categorical value in Eq. 1 is represented by a one-hot vector of length K (i.e., a $\:\times\:$ K), where K is the number of possible classes for that categories^20^. The vector contains zeros in all cells except one with the value 1, which specifies the actual class.

1 $$\:{\left[\varnothing\:\:\right(x\left)\right]}_{k}=1\:\left\{x={c}_{k}\right\},\:\:\:\:\:\:\:\:\:\:\:k=1,\dots\:..,K,\:\:\:\:\:\:\:\:\:\:\:\:\:\:\sum\:_{k}^{K}{\left[\varnothing\:\:\right(x\left)\right]}_{k}=1$$

$\:c=\{\:{c}_{1},\dots\:\dots\:.,{c}_{k}\}$ Is the category set (with size K), x $\:\epsilon$ c is observed category, $\:{\left[{\varnothing}\:\right(x\left)\right]}_{k}\:$denotes the K-th coordinate of the one-hot vector, 1{⋅} is the indicator function (1 if the condition holds, 0 otherwise), n is the number of samples, the one hot block is O $\:\epsilon$ $\:{\left\{\mathrm{0,1}\right\}}^{n\times\:k}$ in Eq. 2.

2 $$\:{O}_{ik}=1\:\{\:{x}_{i}=\:{c}_{k}\}$$

This formulation ensures that the learning model does not assume that “larger numbers are more important,” because simple numerical notations (e.g., “stable = 1, more stable = 2, unstable = 3”) might imply an unreal rank relationship or distance between classes. For example, the value “emerging” is not “more important” than “scheduled” because we write it as 2 instead of 1; they are simply different labels for two classes that should be separated without an imaginary order. In practice, we keep the columns of one-hot values as binary (0/1) without resizing scaling.

#### Mixed-Scaling

**Fig. 2:**
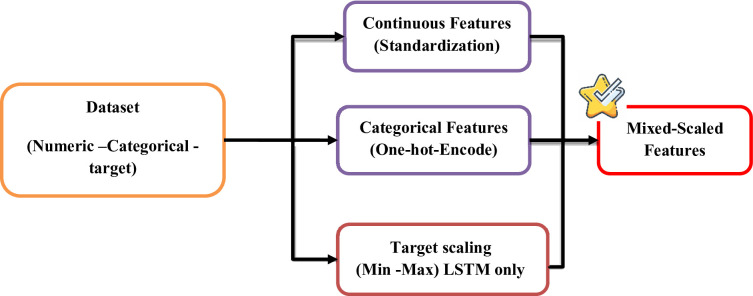
Mixed-Scaling preprocessing strategy used in the proposed ASTP framework

To ensure numerical stability during model training while preserving the semantic meaning of categorical indicators, a mixed-scaling preprocessing strategy is adopted. In this approach, different types of variables are treated according to their statistical characteristics, as illustrated in Fig. 2. The mixed-scaling strategy applies standardization to continuous input features and min–max normalization to the regression target, while leaving the binary/one-hot indicators unchanged. For continuous input features, Z-score standardization is applied. For each continuous feature$\:{\:X}_{j}$, the mean $\:{\mu\:}_{j}$ and stander deviation $\:{\sigma\:}_{j}\:$on the training split only, and transform every sample $\:i$ as in Eq. 3.

3 $$\:{\overline{X}}_{ij}=\frac{{X}_{ij}-{\mu\:}_{j}}{{\sigma\:}_{j}}$$

This centers on features (mean$\:\cong\:0$) and scales their variance ($\:\cong\:1$), improving numerical stability for gradient-based optimization. Binary and one-hot features remain in {0, 1} without scaling to preserve their presence/absence semantics. Min–Max normalization is applied to the target variable (surgery runtime in minutes). During LSTM training, the target $\:y$(runtime in minutes) is mapped to the range [0, 1] using the minimum $\:{y}_{min}$ and maximum $\:{y}_{max}\:$values computed from the training data in Eq. 4:

4 $$\:{y}_{s}=\frac{y-{y}_{min}}{{y}_{max}-{y}_{min}}\:\:\:\:\:,\:\:\:\:\widehat{y}={y}_{min}+{\:\widehat{y}}_{s}({y}_{max}-{y}_{main})$$

This scaling stabilizes the optimization process and ensures that the loss function operates within a consistent range. After inference, the predicted values are inverse-transformed to minutes for reporting and interpretation. In the Random Forest pipeline, the target is left in minutes, tree-based ensemble models do not require scaling of the target variable.

#### Experiencing The Impact of The Ranking/Importance Related to each Feature

At this stage, we employ two models—a single-layer long short-term memory (LSTM) network and a random forest to balance expressive power with robustness on tabular data. The LSTM acts as a gated feature mixer, even with a single time step, capturing subtle nonlinear interactions while maintaining stable gradients through its cell and hidden states with a tanh nonlinearity. A compact dense head maps the LSTM representation to the scalar target. In contrast, the random forest provides a strong, noise-tolerant baseline that requires minimal preprocessing, naturally handles mixed numeric/one-hot inputs, and affords straightforward interpretation via permutation importance. We use both learners as a combined robustness check: their complementary inductive biases probe the same signal from different angles. Agreement between their attributions SHAP for the LSTM and permutation importance (MAE) for the random forest strengthens confidence in the learned relationships, whereas discrepancies highlight features or regimes that merit closer inspection. Where appropriate, a simple averaging ensemble can further reduce variance and improve MAE. In Algorithm 1, the steps of the feature importance (RF permutation and LSTM–SHAP) used to quantify surgical-time drivers are provided.

#### Long Short-Term Memory (LSTM)

Recurrent neural networks (RNNs) designed to learn long-term temporal dependencies in sequential data. Traditional RNNs struggle to retain information over long time periods because gradients either disappear or explode during backpropagation^21^. LSTM networks address this problem by adding an explicit memory cell and a set of gates that regulate the flow of information, allowing for a stable distribution of values over longer time periods. An LSTM processes a sequence step by step while maintaining two internal states: a cell state $\:{c}_{t}$that carries long-term information, and a hidden state $\:{h}_{t}$ that provides the current output. Three gates (input, forget, and output) control writing, erasing, and reading from the cell. The gates are parameterized by sigmoid (values in [0, 1]) so the model can learn how much new information to admit, how much past information to keep, and how much of the updated memory to expose. A hyperbolic tangent (tanh) nonlinearity proposes new content for the cell and squashes the exposed state, keeping activations bounded and gradients more stable.

**Fig. 3:**
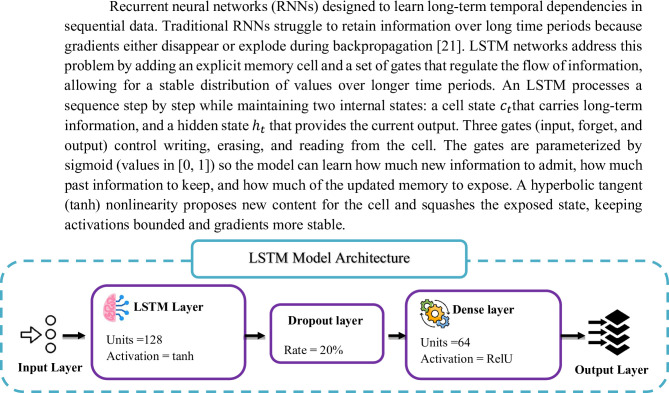
LSTM model Architecture in ASTP

In Fig. 3. Show LSTM component used in our model. The LSTM operates on tabular inputs reshaped to a single time step (N, 1, d) maintaining a cell state $\:{c}_{t}\:$and a hidden state $\:{h}_{t}$ while three sigmoid gates (input, forget, output) regulate writing, retention, and exposure of information. The tanh nonlinearity proposes new content and bounds activations, stabilizing gradients; with sequence length one, the forget gate plays a minor role, whereas the input and output gates act as a sample-specific soft mask over the feature-driven proposal. We use a single-layer LSTM (u$\:\:\in\:\left\{\mathrm{96,128}\right\},tanh$) followed by dropout (p ∈ {0.10, 0.20}) and a compact dense head (m $\:\:\in\:${32, 64}, ReLU), ending with a linear output (Dense (1)) that produces a scalar estimate of time. The target is min–max scaled on the training split and predictions are inverted back to minutes for reporting. After training the LSTM model, SHAP is applied on the validation set to explain how each input feature contributes to the predicted surgical duration. For each validation sample, SHAP assigns an additional contribution value to every feature. These SHAP values are then rescaled by the target range so that the contribution of each feature is expressed directly in minutes. Finally, the overall significance of each feature is calculated as the average of the absolute SHAP value across all validation samples^22^.

### Random forest

Random forests are ensembles of decision trees trained on resampled data, with attribute subsamples taken at each split to separate the trees and reduce variance^23^. For an input x, the forest expectation is the mean of the T trees in Eq. 5.

5 $$\:\widehat{y}\left(x\right)=\frac{1}{T}\:\sum\:_{t=1}^{T}{f}_{t}\left(x\right)$$

Where $\:{f}_{t}$ is the prediction of tree$\:\:t$, In Random forests the output is reported directly in minutes (no target scaling) such as lstm.

**Figure:**
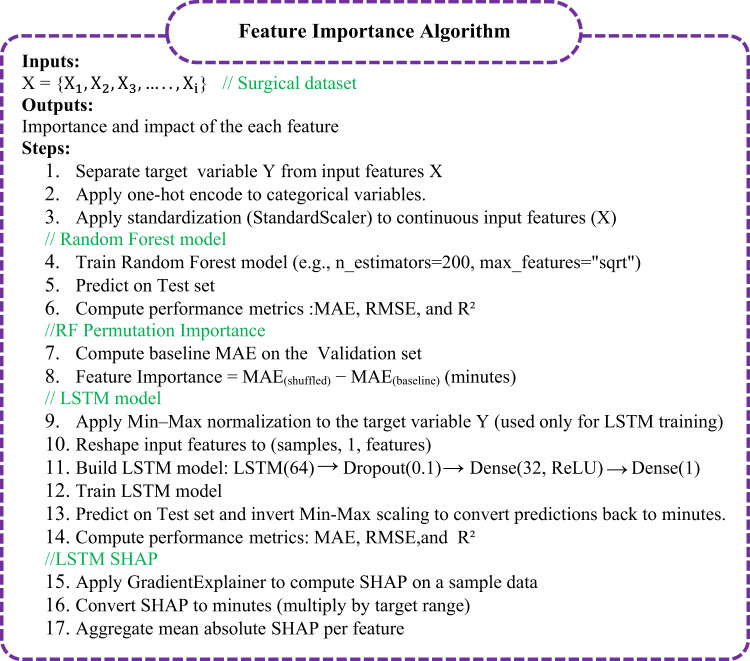
Algorithm 1: Feature Importance for Surgical Time Prediction (RF Permutation + LSTM SHAP)

Therefore, in parallel, a random forest model is trained on the same input variables, and the importance of its features is estimated using the permutation importance. In this method, the values of one feature are randomly rearranged while keeping the other features unchanged, and the resulting rise in the mean absolute error is recorded as ΔMAE. A larger positive value for ΔMAE indicates that the feature is more influential. Two techniques methods are used together to verify the robustness of the results. If both methods produce a similar ranking, confidence in the feature ranking is strengthened. If differences emerge, this indicates that some variables may behave differently under linear and nonlinear interactions, thus requiring further investigation. The final ranked feature list is used in the next stage, where the HGBR model is evaluated using the TOP-K strategy. Therefore, only the LSTM+SHAP model is used for feature ranking.

### Prediction layer in ASTP

In the prediction layer, the features are listed in the order they were taken in the previous Feature Importance and Feature Ranking stage, and we use Histogram Gradient Boosting Regression for prediction. Histogram Gradient Boosting Regression (HGBR) is a gradient-boosted decision tree model that speeds up partition searches by quantizing continuous features across a fixed number of bins and evaluating partitions at bin boundaries rather than all unique values^24^.

This histogram approximation significantly reduces memory consumption and computational costs while preserving the expressiveness of boosted trees. On structured (tabular) data, HGBR typically achieves high accuracy by capturing nonlinear and feature interactions with minimal feature engineering and without strict input scaling forecasting. The HGBR follows a four-stage mechanism. (1) Histogram binning: continuous features are quantized into a fixed number of bins learned during training. This turns split evaluation from sample-level scans into bin-level scans, improving cache locality and runtime while retaining the expressiveness of boosted trees. (2) Split finding: for each node, prefix-sum scans over the per-bin (and per-feature) aggregated gradients identify the threshold that maximizes loss reduction (gain). (3) Gradient boosting loop: shallow regression trees are fitted to the negative gradients (pseudo-residuals) and added with shrinkage; we use L1 loss with leaf-value smoothing (L2) and early stopping for regularization. (4) Output ensemble: multiple seed models are averaged in log space at inference time (equivalent to a geometric mean on the original scale), yielding a stable predictor. In our pipeline, this module generates log-space predictions that are inverted to minutes for reporting.

In our strategy, we use the Histogram Gradient Boosting Regression (HGBR) model as the main tabular updater to predict the surgical duration (in minutes). The target variable is expressed in minutes and, on the training split only, we apply target preprocessing Winsorization using an IQR rule constrained by the 1st/99th percentiles, followed by log1p transform with an optional non-negative shift s $\:\ge\:0$. At inference, predictions are mapped back to minutes via Eq. 6.

6 $$\:\widehat{y}=\mathrm{max}\:\{\:0,\:expm1\left(\widehat{z}\right)\--\mathrm{s}\:\}\:\:$$

**Fig. 4:**
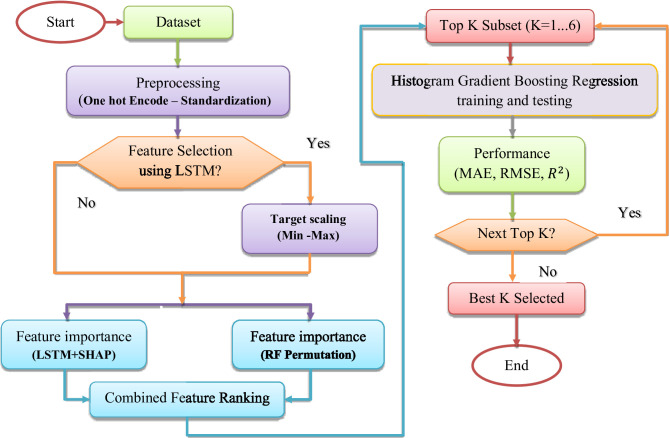
Flowchart of the proposed Accurate Surgery Time Prediction (ASTP) Steps.

And all metrics are reported in minutes. We use a priority-ordered feature set from feature ranking stage. The HGBR configuration is tuned for the tabular performance, specifying the loss learning rate, maximum product, maximum leaf nodes, minimum leaf samples, l2 normalization, and maximum bins, with an early internal stop (validation rate = 0.20). Mechanistically, HGBR speeds up boosting by quantizing each feature into up to B bins (we use B $\:\le\:$ 162) and evaluating split candidates at bin boundaries using bin-level prefix-sum statistics; at each round a shallow tree is fitted to the pseudo-residuals and added with shrinkage. For stability, we train three seed models and average predictions in log space (geometric mean on the original scale). This design yields a fast, robust, and accurate baseline for structured surgical-time forecasting. All steps of Algorithm 2 are explained. Figure 4 illustrate the complete flowchart of the proposed strategy.

**Figure:**
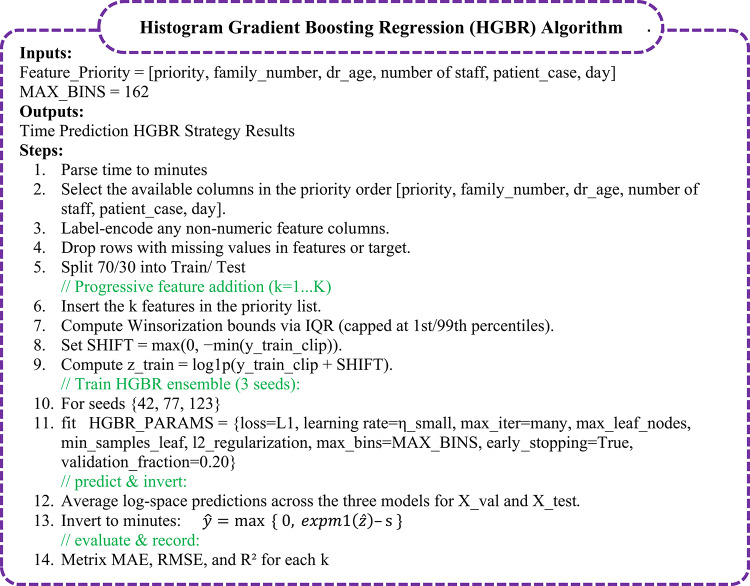
Algorithm 2: HGBR for surgical-time prediction

### Evaluation and results

**Fig. 5:**
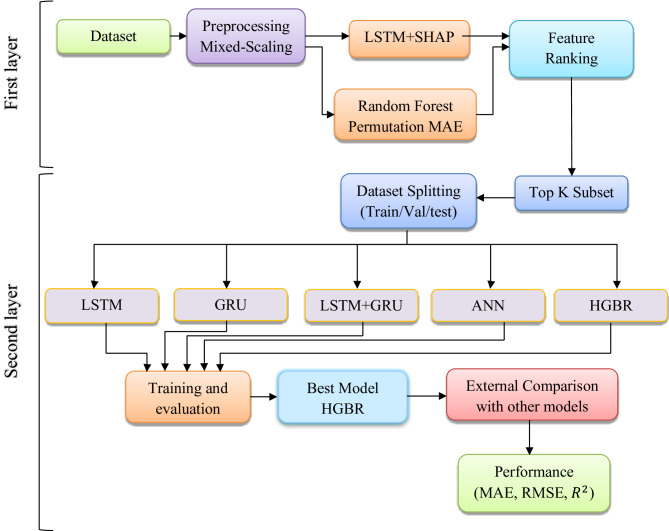
Overall workflow for the proposed Accurate Surgical Time Prediction (ASTP) with all comparison methods.

This section presents the experimental evaluation of the proposed ASTP framework, including the results obtained in each layer and their application to the surgical dataset. The experiments were performed on Windows 11 using the Python programming language. The main libraries used were NumPy, Pandas, SHAP, keras, and Scikit-Learn. The computing environment consisted of an Intel Core i7 processor with 16 GB RAM. Figure 5 illustrates the overall workflow of the proposed Accurate Surgery Time Prediction (ASTP) with all comparisons, which is divided into two layers: Layer 1 for feature ranking and TOP-K extraction, and Layer 2 for model training, evaluation, and reporting.

### Dataset

The experiments were applied on two datasets. The first was the Operating room dataset from Nile Hospital^25^, while the second was The Medical Informatics Operating Room Vitals and Events Repository (MOVER) dataset^28^.

#### Operating room data from Nile Hospital

Dataset that contains all surgical operations for the period between December 1, 2023, and December 31, 2023. It consists of seven columns that provide daily information as follows: (1) day, (2) priority, (3) time, (4) patient case, (5) number of staff, (6) dr_age, and (7) family number. The Staff column represents the number of medical staff in the operating room, excluding surgeons, and the Patient Condition column indicates the stability of the patient’s condition and ensures readiness to enter the operating room. The priority column expresses emergency cases and their priority in performing surgery. The dataset contains 471 samples and seven columns. Samples from the modified dataset are shown in Table 2.

**Table 2:** Samples from the used dataset containing seven features before preprocessing.

day	priority	time	patient case	number of staff	dr_age	family number
1-Dec	5	90 min	Stable	6	60	3
	1	35 min	Stable	8	55	2
	2	20 min	Unstable	5	55	1
	7	75 min	Stable	8	55	2
	7	75 min	Stable	9	55	2
	7	75 min	Stable	7	55	2
	7	75 min	Stable	8	50	5
	7	90 min	Stable	9	48	4
	2	90 min	Unstable	5	38	1
2-Dec	4	60 min	Stable	7	40	1
	7	30 min	Unstable	8	40	2
	7	60 min	Stable	9	40	3
	7	60 min	Stable	7	40	3
	5	60 min	Stable	7	40	4
	3	60 min	Stable	8	40	2
	4	45 min	Stable	9	38	2
	2	45 min	Stable	7	40	4
	7	60 min	Unstable	9	45	3
	7	40 min	Stable	8	45	3
	7	60 min	Stable	9	60	2
	7	30 min	stable	8	60	3
	7	60 min	Stable	8	48	2
	7	30 min	stable	6	48	2
	7	60 min	Stable	8	48	2
	7	45 min	Stable	9	48	2
	7	30 min	stable	8	48	3
	7	20 min	Stable	7	48	3
	7	30 min	Unstable	8	48	2
	7	30 min	Stable	9	50	3
	7	30 min	Stable	7	50	3
3-Dec	7	30 min	Unstable	7	60	1
	7	45 min	Stable	8	60	2
	7	45 min	Unstable	8	60	2
	7	45 min	Stable	9	60	2
	7	30 min	Stable	7	60	2

The raw data cannot be used directly for prediction because the input variables are represented in heterogeneous formats, including categorical labels, textual time expressions, and numerical values. Therefore, a preprocessing step is required to convert the raw data into a suitable numerical representation for model development. This step converts the date column to a number representing a day during the workweek or weekend, where 0 represents a weekend day and 1 represents a day during the workweek. The patient status column is converted to a number representing steady state with 1 and unstable state with 0. The time column is converted to minutes and scaled using Min-Max, while the feature columns, family number, number of staff and priority are scaled using StandardScaler. Table 3 provides a transformed representation of the dataset after preprocessing. However, in the final ASTP framework, preprocessing is applied in a model-specific manner. Target scaling is used only in the LSTM branch, whereas HGBR and Random Forest operate directly on the target in minutes after their respective preprocessing steps.

**Table 3:** Samples from the used dataset containing seven features after preprocessing.

day	priority	time	patient case	number of staff	dr_age	family number
0	−0.32536	0.318182	1	0.880257	2.239661	0.388964
0	−2.24692	0.181818	1	0.37655	1.453024	−0.70152
0	−1.76653	0	0	−1.13457	1.453024	−1.79201
0	0.635421	0.25	1	0.37655	1.453024	−0.70152
0	0.635421	0.25	1	0.880257	1.453024	−0.70152
0	0.635421	0.25	1	−0.12716	1.453024	−0.70152
0	0.635421	0.318182	1	0.37655	0.666387	2.56994
0	0.635421	0.318182	1	0.880257	0.351732	1.479452
0	−1.76653	0.045455	0	−1.13457	−1.22154	−1.79201
1	−0.80575	0	1	−0.12716	−0.90689	−1.79201
1	0.635421	0.045455	0	0.37655	−0.90689	−0.70152
1	0.635421	0.181818	1	0.880257	−0.90689	0.388964
1	0.635421	0.181818	1	−0.12716	−0.90689	0.388964
1	−0.32536	0.181818	1	−0.12716	−0.90689	1.479452
1	−1.28614	0.181818	1	0.37655	−0.90689	−0.70152
1	−0.80575	0.113636	1	0.880257	−1.22154	−0.70152
1	−1.76653	0.090909	1	−0.12716	−0.90689	1.479452
1	0.635421	0.181818	0	0.880257	−0.12025	0.388964
1	0.635421	0.181818	1	0.37655	−0.12025	0.388964
1	0.635421	0.045455	1	0.880257	2.239661	−0.70152
1	0.635421	0.181818	1	0.37655	2.239661	0.388964
1	0.635421	0.045455	1	0.37655	0.351732	−0.70152
1	0.635421	0.181818	1	−0.63086	0.351732	−0.70152
1	0.635421	0.181818	1	0.37655	0.351732	−0.70152
1	0.635421	0.113636	1	0.880257	0.351732	−0.70152
1	0.635421	0.045455	1	0.37655	0.351732	0.388964
1	0.635421	0.113636	1	−0.12716	0.351732	0.388964
1	−0.80575	0	1	−0.12716	−0.90689	−1.79201
1	0.635421	0.045455	0	0.37655	−0.90689	−0.70152
1	0.635421	0.181818	1	0.880257	−0.90689	0.388964
1	0.635421	0.181818	1	−0.12716	−0.90689	0.388964
1	−0.32536	0.181818	1	−0.12716	−0.90689	1.479452
1	−1.28614	0.181818	1	0.37655	−0.90689	−0.70152
1	−0.80575	0.113636	1	0.880257	−1.22154	−0.70152
1	−1.76653	0.090909	1	−0.12716	−0.90689	1.479452

#### MOVER dataset

The Medical Informatics Operating Room Vitals and Events Repository (MOVER) is a perioperative dataset containing structured electronic health records and operating room information for surgical cases. In this study, the dataset used contained 39,381 samples. After preprocessing and feature engineering, the dataset was represented using 14 columns, including 13 input features and one target output, which was surgery_duration_min. Input variables included: sex, primary_anes_type_nm, asa_rating, patient_class_nm, height_cm, weight_kg, bmi, surgery_weekday, surgery_month, scheduled_hour, admit_to_or_hours, procedure_grp, and dx_grp. Several variables were extracted from the original dataset, such as height_cm, weight_kg, bmi, surgery_weekday, surgery_month, scheduled_hour, and admit_to_or_hours, while high-cardinality variables were grouped into procedure_grp and dx_grp. Thus, the final modified dataset used in this work contained 39,381 samples with 13 predictive features and one target variable.

### Experimental setup

The principal experimental settings and model parameters adopted in our strategy are consolidated in Table 4. The table summarizes the data partitioning strategy, preprocessing procedures, model-specific hyperparameters, training configuration, and evaluation metrics used throughout the ASTP framework.

**Table 4:** Main Experimental Settings and Model Parameters Used in the ASTP Strategy.

Data split		Parameter	Value
		Train/test split	70%/30%
Preprocessing		Categorical handling	Encoding applied to categorical variables
		Continuous inputs	Standardization
		LSTM target scaling	Min–Max normalization on train only
		HGBR target preprocessing	Winsorization + log1p
Feature ranking	LSTM	Hidden units	u∈{96,128}
		Dropout	p∈{0.10,0.20}
		Dense head	m∈{32,64}, ReLU
	Random Forest	Importance method	Permutation importance
HGBR		Loss	absolute_error
		Learning rate	0.034828
		Max iterations	1340
		Max leaf nodes	533
		Min samples per leaf	4
		Max bins	162
Prediction strategy		Final model	HGBR with TOP-K subsets

The ASTP framework was evaluated using a fixed training-testing split for each dataset, where 70% of the cases were used for training and 30% were reserved for final testing. Therefore, all final performance results reported in the following subsections correspond to unseen real cases that were not used during model development.

### Performance metrics

The performance metrics used are (1) mean absolute error (MAE), (2) root mean square error (RMSE), and (3) coefficient of determination (R²), which are calculated to evaluate the prediction accuracy of the dataset for surgical time estimation.

#### Mean absolute error

MAE is a widely used assessment metric that assesses the average magnitude of errors between predicted values.$\:\:{\widehat{y}}_{i}$ And the actual observed values $\:{y}_{i}$ without contemplating their destination. It gives an understandable measure of the average distance between forecasts and true values, given in the same unit as the target variable.

$${\rm MAE}=\:\frac{1}{n}\:\sum\:_{i=1}^{n}\:\left|{y}_{i}-\right.\left.{\widehat{y}}_{i}\right|$$

Where n total number of observed,$\:\:{y}_{i}$ is actual value, $\:\widehat{y}\:\:$Is predicted value, and $\:\left|{y}_{i}-\right.\left.{\widehat{y}}_{i}\right|$ is absolute error for observation $\:i$.

#### Root Mean Square Error

RMSE calculates the square root of the average squared difference between the expected values.$\:\widehat{y}\:$And the actual observed values $\:{y}_{i}$. Unlike MAE, RMSE penalizes greater error more severely owing to squaring, making it susceptible to outliers.

$${\rm RMSE} =\:\sqrt{\frac{1}{n}\sum\:_{i=1}^{n}{({y}_{i}-{\widehat{y}}_{i})}^{2}\:}$$

Where n total number of observed,$\:\:{y}_{i}$ is actual value, and $\:\widehat{y}\:\:$Is predicted value.

#### Coefficient of Determination

R² calculates the fraction of variation in real values $\:{y}_{i}.$ this is explained by the projected values. $\:{\widehat{y}}_{i}$ It indicates the model’s quality of fit.

$${\rm R^{2}}=1-\:\frac{\sum\:_{i=1}^{n}{({y}_{i}-{\widehat{y}}_{i})}^{2}}{\sum\:_{i=1}^{n}{({y}_{i}-{\stackrel{-}{y}}_{i})}^{2}}$$.

Where $\:\:{y}_{i}$is actual value, $\:\widehat{y}\:\:$Is predicted value, and $\:{\stackrel{-}{y}}_{i}$mean of actual values.

### Feature importance and Ranking on Nile Hospital dataset

The current subsection presents the feature-importance analysis obtained using the two proposed method, LSTM+SHAP and Random Forest permutation importance on the dataset.

#### Evaluating The Performance of LSTM Along with Applying SHAP for Explanation

**Fig. 6:**
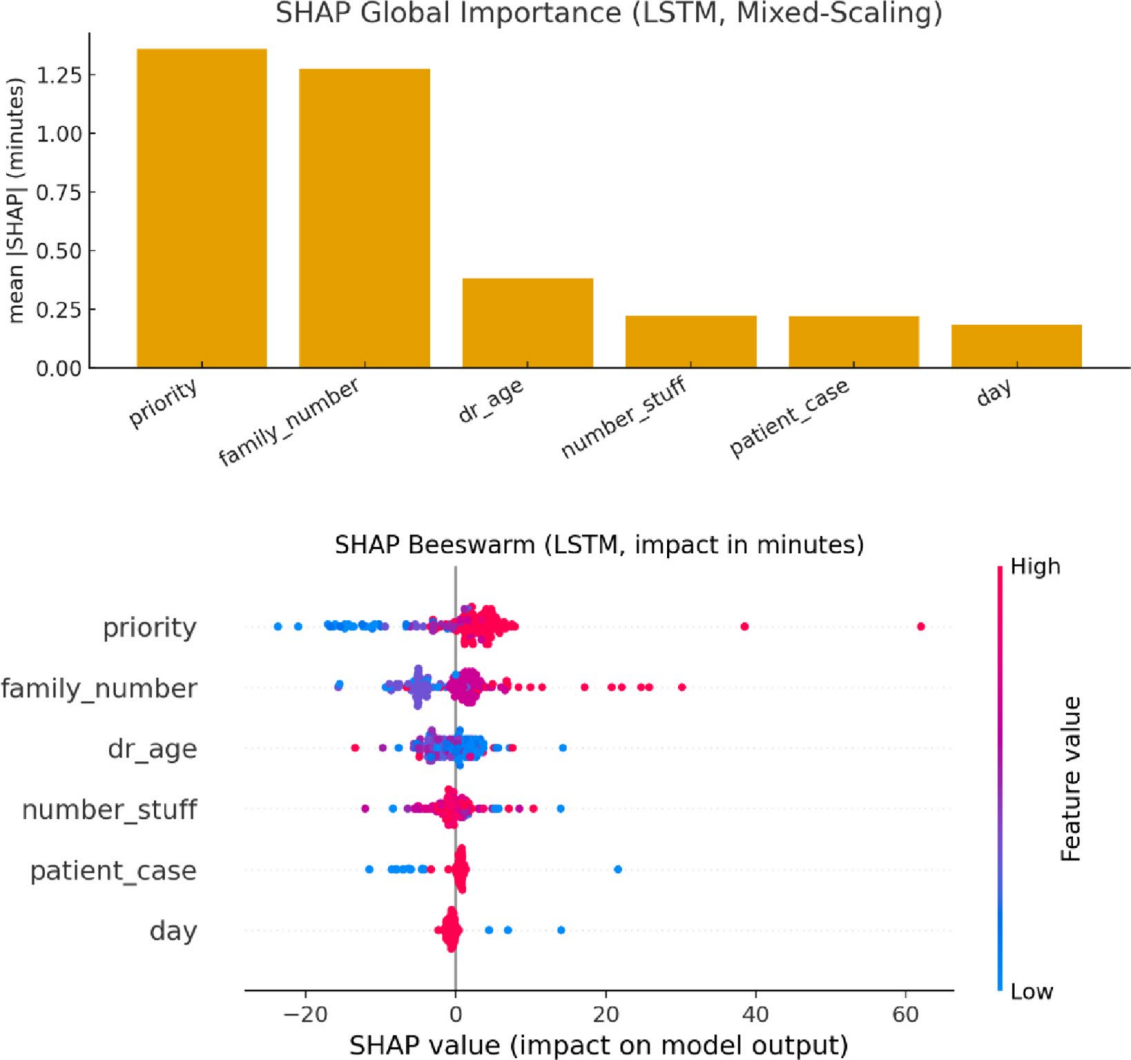
SHAP values for each feature based on the mean absolute SHAP value for the LSTM surgical-time predictor.

In this section, we describe the implementation of LSTM with Explainable AI (XAI) using SHAP to calculate feature importance in the dataset. The goal is to determine the impact of each feature on the output (time of surgical operations) and assess their relative importance. Figure 6 illustrates the significance of SHAP-based features for the LSTM model. The distribution of SHAP values indicates that priority is the most influential feature, followed by family_number and dr_age. Number of staff and patient_case have less influence, while day shows the weakest overall effect. This ranking confirms that the predicted surgical time is depends primarily on information related to priority and procedure.

Table 5 shows the ranking of feature importance based on SHAP values using the mean absolute SHAP value. Higher values indicate the feature’s importance in the model’s decision-making process. Thus, in the mean absolute SHAP value, the priority feature has the highest value, with the features ranked as follows: (priority, Family number, dr_age, number of staff, patient case, and day).

**Table 5:** Feature importance values using Mean Absolute SHAP value.

feature	mean_abs_shap_min
priority	1.3607
family_number	1.2757
dr_age	0.3836
number of staff	0.2227
patient_case	0.2196
day	0.1805

#### Evaluating the performance of randomforest with applying permutation importance for explanation

In the Random Forest branch, feature importance is measured using permutation importance. Each feature is randomly while the remaining features are kept unchanged, and the corresponding increase in MAE is recorded as ΔMAE=MAE_permuted_ −MAE_baseline_. Larger positive ΔMAE values indicate greater feature importance, while values close to zero indicate limited predictive contribution.

The importance of the permutations was calculated using a cross-validation split of a modified random forest. The mean baseline error (MAE) was 7.35 min. For each feature, we calculated the mean ΔMAE across repeated shuffling. Table 6 illustrate the Permutation importance results in terms of ΔMAE.

**Table 6:** Feature importance values using ΔMAE value.

feature	ΔMAE	Baseline MAE	Permuted MAE
priority	5.54	7.35	12.88
family_number	4.28	7.35	11.62
dr_age	3.24	7.35	10.59
number of staff	1.68	7.35	9.02
patient_case	0.23	7.35	7.57
day	−0.07	7.35	7.27

**Fig. 7:**
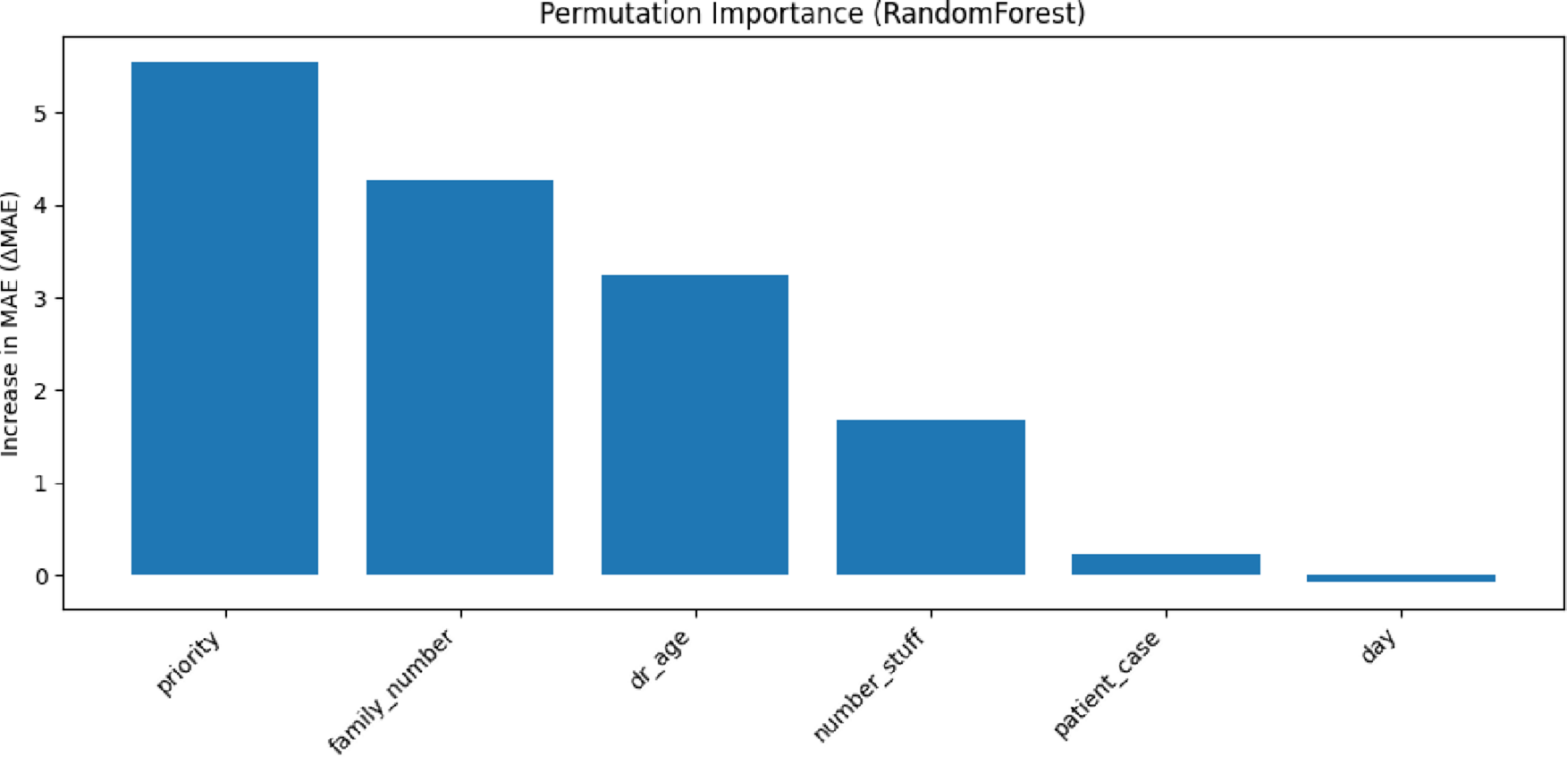
importance of permutations (ΔMAE, in minutes).

Figure 7 confirms the same ranking trend obtained from SHAP. Priority and family_number cause the largest increase in error after permutation, followed by dr_age and number of staff, while patient_case and day contribute only marginally. This suggests that the day variable adds little predictive information to the current dataset.

The LSTM with SHAP Implementation and Random Forest with Permutation Importance provided the same results in terms of the importance ranking of the features, but if the results differed, the prediction system would not be able to know the order of features to input into the model. Therefore, the metrics from the two methods were averaged to produce a single metric that was fed into the temporal prediction system. Table 7 displays the results for the average values.

**Table 7:** Feature importance from SHAP vs. permutation ΔMAE and their average score.

feature	mean_abs_shap	ΔMAE	average
priority	1.3607	5.54	3.45035
family_number	1.2757	4.28	2.77785
dr_age	0.3836	3.24	1.8118
number of staff	0.2227	1.68	0.95135
patient_case	0.2196	0.23	0.2248
day	0.1805	−0.07	0.05525

**Fig. 8:**
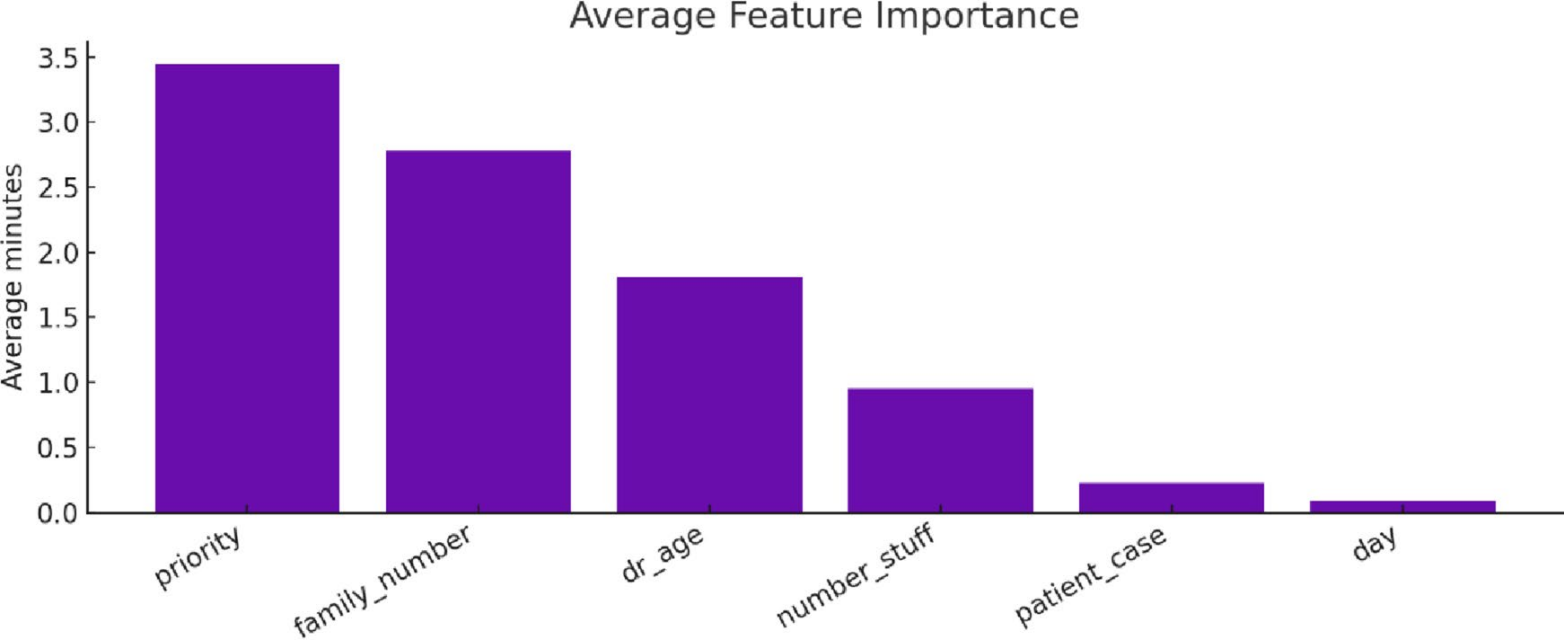
Average feature importance from SHAP and ΔMAE.

In the Fig. 8, the average results for mean_abs_shap and ΔMAE are shown. Were the results show that priority (approximately 3.45, or about 37%) has the most impact; changing/disabling it makes the biggest difference in predicted operation time. family_number (approximately 2.78, or about 30%) is the second most important variable. dr_age (approximately 1.81, or about 19%) and number of staff (approximately 0.95, or about 10%) has a medium impact. Patient (approximately 0.22, or about 2%) has a weak impact. day (approximately 0.06, or less than 1%) has negligible impact.

### Ablation study to reduce features in the proposed ASTP framework on nile hospital dataset

To further emphasize the importance of the feature selection phase in the proposed ASTP framework, an analytical study was conducted using the HGBR model in two cases: (i) input of all six available features, (ii) input of the top five ranked features, and (iii) input of the top four ranked features, derived from the combined LSTM+SHAP and Random Forest permutation-importance analysis. The ranking results from both methods were highly consistent, with priority, family_number, dr_age, and number of staff identified as the most influential features. The ablation results show that the reduced model with four features achieved a lower mean absolute error (MAE) (8.8857 min) than the complete model with six features (9.0512 min), indicating improved feature efficiency and more accurate predictive representation. On the other hand, the five- and six-feature configurations maintained slightly better root mean square error (RMSE) and R² values than the four-feature configuration, indicating a trade-off between minimizing input dimensions and preserving overall variance fit. Overall, these results confirm the effectiveness of the feature-ranking and TOP- K stages in the ASTP algorithm are effective for minimizing input space while maintaining competitive predictive performance. Table 8 summarizes the effect of feature reduction on the final prediction phase of HGBR.

**Table 8:** Ablation study of feature reduction in the proposed ASTP framework.

HGBR(full-feature(	Features used	MAE	RMSE	*R* ^2^
	6 features	9.0512	19.3574	0.2725
HGBR (intermediate-feature)	Top 5 ranked features	8.9389	19.3543	0.2727
HGBR (reduced-feature)	Top 4 ranked features	8.8857	19.5192	0.2603

### In-depth evaluation of the HGBR strategy to assess performance effectiveness on nile hospital dataset

From the previous layer, we obtained the following attribute order (priority, family_number, dr_ age, number of staff, patient_case, and day). We apply the model to a set of TOP K, where each K feature is added in the order we obtained from the previous layer. For example, TOP K 1 represents the set consisting of only the priority feature, while TOP K 2 represents the set consisting of priority and family_number. This continues with the remaining sets until TOP K 6, which contains all the features. Table 9 summarizes the prediction performance of the HGBR, ANN, LSTM, GRU, and LSTM + GRU hybrid models across increasing subsets of the TOP-K features. Overall, the HGBR model achieved the lowest mean absolute error (MAE) using the top four ranked features, while the ANN model performed best using all six features. The iterative models (LSTM, GRU, and the hybrid) consistently produced higher errors than the HGBR model across the evaluated subsets. These results suggest that the prediction layer based on the HGBR model is better suited to the current scheduled surgery time dataset.

**Table 9:** The results of the HGBR and other machine learning models with different feature subsets (TOP K) on the surgical time prediction Nile Hospital dataset.

TOP K	Features	Models	MAE	RMSE	$\:{\boldsymbol{R}}^{2}$
1	• priority	HGBR	10.5970	20.2082	0.2071
		ANN	13.4008	20.0643	0.2184
		LSTM	13.5027	21.6060	0.0936
		GRU	13.4146	21.5717	0.0965
		LSTM + GRU	14.2266	22.1202	0.0500
2	• priority • family_number	HGBR	9.2032	19.0879	0.2926
		ANN	10.6425	18.9794	0.3006
		LSTM	11.9034	20.0850	0.2167
		GRU	11.7373	19.8910	0.2318
		LSTM + GRU	12.0184	20.1225	0.2138
3	• priority • family_number • dr_age	HGBR	9.0708	20.3573	0.1954
		ANN	11.0391	20.1366	0.2127
		LSTM	12.2478	20.3723	0.1942
		GRU	12.3939	20.4998	0.1841
		LSTM + GRU	13.0909	20.8790	0.1536
4	• priority • family_number • dr_age • number of staff	HGBR	8.8857	19.5192	0.2603
		ANN	10.6233	19.8060	0.2384
		LSTM	12.3418	20.4425	0.1886
		GRU	12.0412	20.2012	0.2077
		LSTM + GRU	13.0429	20.8664	0.1546
5	• priority • family_number • dr_age • number of staff • patient_case	HGBR	8.9389	19.3543	0.2727
		ANN	10.3207	19.1051	0.2913
		LSTM	12.2317	20.2734	0.2020
		GRU	12.1265	20.3900	0.1928
		LSTM + GRU	13.1159	21.0785	0.1373
6	• priority • family_number • dr_age • number of staff • patient_case • day	HGBR	9.0512	19.3574	0.2725
		ANN	9.9891	18.6954	0.3214
		LSTM	12.1691	20.2299	0.2054
		GRU	12.1165	20.3876	0.1930
		LSTM + GRU	13.5552	21.4625	0.1056

**Fig. 9:**
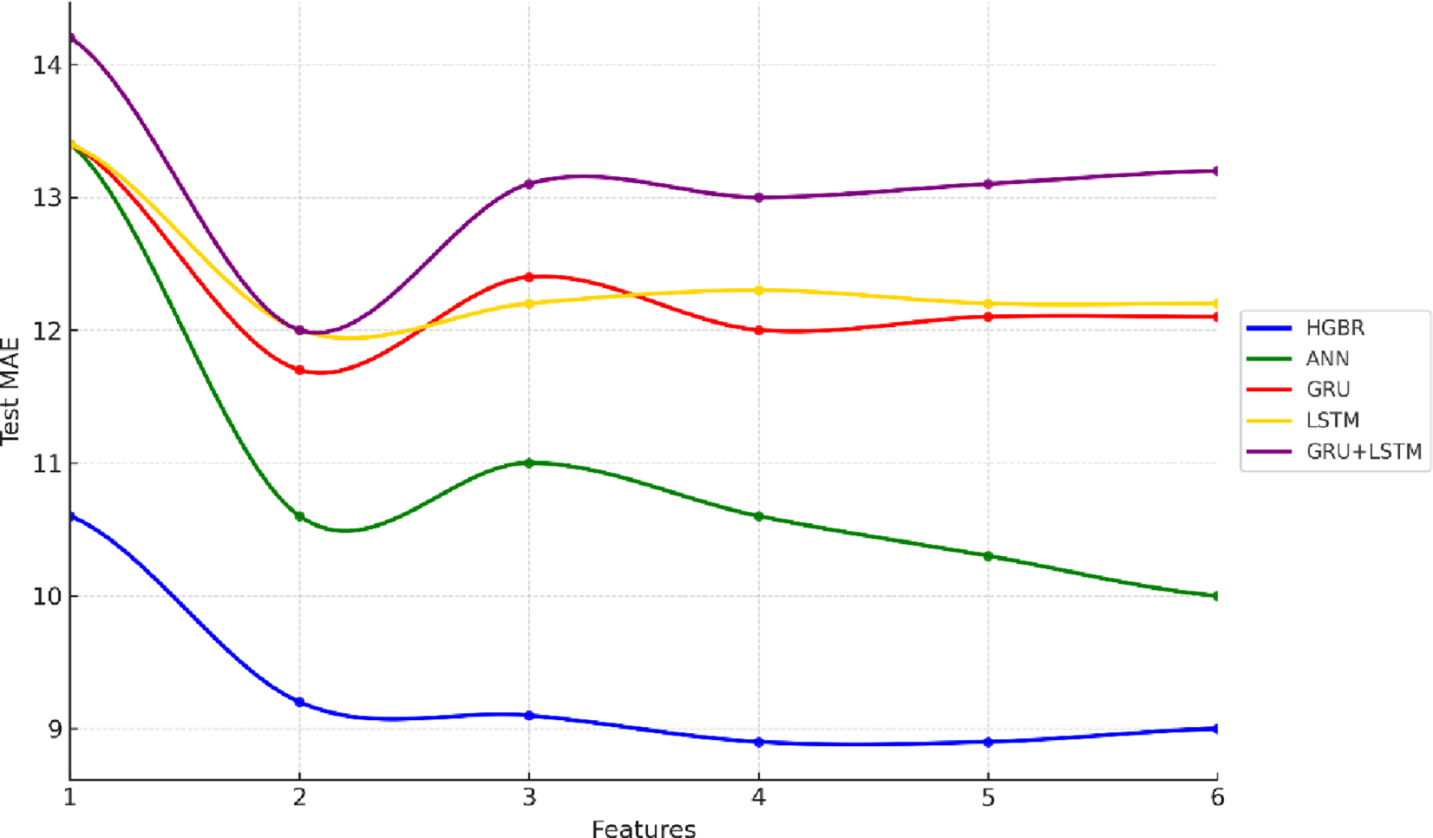
MAE performance of different models (HGBR, ANN, GRU, LSTM, and LSTM + GRU) across incremental feature subsets (TOP K) on Nile Hospital dataset.

Table 9 presents the best values achieved by HGBR, ANN, LSTM, GRU, and hybrid(LSTM + GRU). HGBR performed the best, reaching its lowest error with only four features. LSTM, GRU, and hybrid (LSTM + GRU) achieved their lowest values with two features; however, these values were still higher than those of HGBR. As the number of features increased, the values continued to rise, while ANN performed better than LSTM, GRU, and hybrid (LSTM + GRU), but with six features, as illustrated in Fig. 9.

**Table 10:** Best performance results for each model in terms of MAE, RMSE, and R².

	Number Feature	MAE	RMSE	$\:{R}^{2}$
HGBR	4	8.8857	19.5192	0.2603
ANN	6	9.9906	18.6985	0.3211
LSTM	2	11.6997	20.0000	0.2234
GRU	2	11.7373	19.8910	0.2318
LSTM + GRU	2	12.0184	20.1225	0.2138

**Fig. 10:**
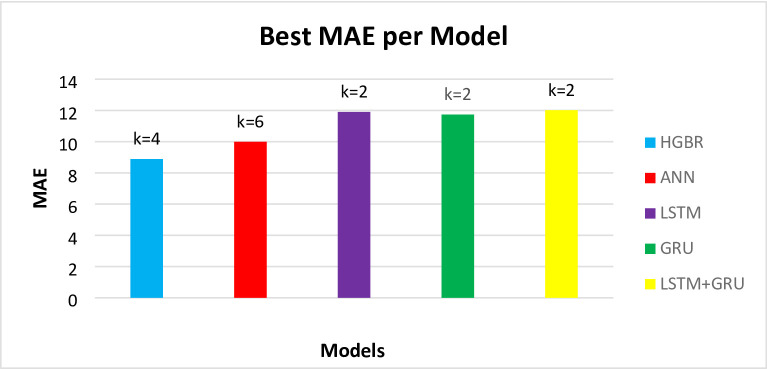
Best MAE per model with the corresponding number of features (K).

**Fig. 11:**
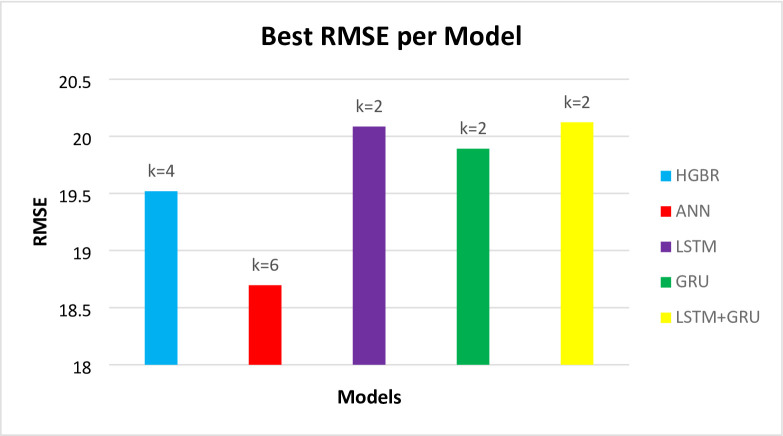
Best RMSE per model with the corresponding number of features (K).

**Fig. 12:**
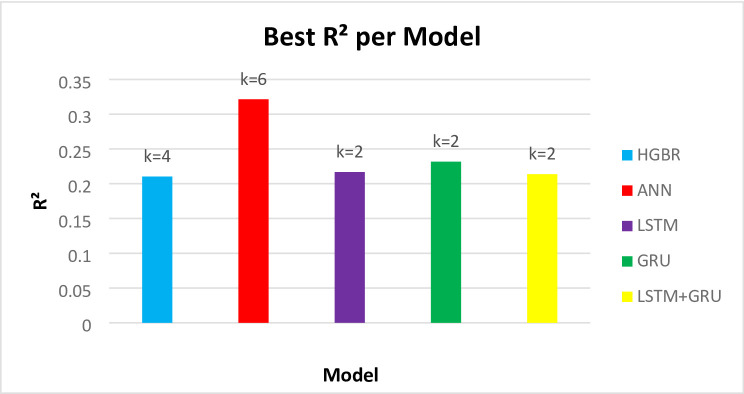
Best R² per model with the corresponding number of features (K).

The ASTP framework was evaluated as an integrated pipeline on a 30% reserved test subset. The best overall performance was achieved by the HGBR-based ASTP configuration using the top four ranked features, resulting in a mean absolute error (MAE) of 8.89 min, a root mean square error (RMSE) of 19.5 min, and a coefficient of determination (R²) of 0.26. This indicates that on average, final expectancy of ASTP differs from the actual surgery duration by approximately 8.89 min in real, non-visualized test cases. Figures 10 and 11, and 12 show a comparison of the best performance of each model across different metrics, demonstrating that HGBR outperforms the other systems.

### Statistical Significance Analysis on Nile Hospital dataset

To assess whether the observed differences between the comparative models were statistically significant, a non-parametric statistical analysis of the absolute prediction errors was performed on the same reserved test set. Specifically, a pair-permutation test was applied, and a 95% Bootstrap confidence interval was calculated for the mean absolute error difference (MAE). The MAE difference was defined as:

ΔMAE = MAE _HGBR_ - MAE _compared model_.

where a negative value indicates that HGBR achieved a lower prediction error than the comparative model.

**Table 11:** Statistical significance analysis of HGBR against the compared models.

Comparison	MAE of HGBR (min)	MAE of Compared Model (min)	ΔMAE (HGBR-Model)	*p*-value	95% CI	Interpretation
HGBR vs. ANN	8.8857	9.9906	−1.1049	0.0084	[−1.9152, −0.2889]	HGBR significantly better
HGBR vs. LSTM	8.8857	11.6997	−2.8140	0.0001	[−3.6093, −2.0243]	HGBR significantly better
HGBR vs. GRU	8.8857	11.7373	−2.8516	0.0001	[−3.7002, −2.0308]	HGBR significantly better
HGBR vs. Hybrid	8.8857	12.0184	−3.1327	0.0001	[−3.9444, −2.3302]	HGBR significantly better

In Table 11 statistical significance analysis confirmed that the HGBR-based ASTP model significantly outperformed all comparable models. Compared to the Artificial Neural Network (ANN), the mean absolute error (MAE) difference was − 1.1049 min, with a p-value of 0.0084 and a 95% confidence interval (CI) of −1.9152 to −0.2889. Compared to the LSTM, the MAE difference was − 2.8140 min, with a p-value of 0.0001 and a 95% CI of −3.6093 to −2.0243. Compared to the GRU, the MAE difference was − 2.8516 min, with a p-value of 0.0001 and a 95% CI of −3.7002 to −2.0308. In comparison with the LSTM + GRU hybrid model, the mean absolute error (MAE) difference was − 3.1327 min, with a p-value of 0.0001 and a 95% confidence interval ranging from − 3.9444 to −2.3302. Since all p-values were less than 0.05 and none of the confidence intervals include zero, the superiority of the HGBR model is statistically significant. These results provide formal statistical evidence that the reduction in prediction error achieved by HGBR is not due to random variance in the test subset, but reflects a consistent predictive advantage of the current structured surgical time dataset.

From the results shown in Table 10 and the statistical significance analysis in Table 11, HGBR provided the best results in the previous comparison, so it was compared with other research works.

### Evaluation on the MOVER Dataset

Table 12 presents the prediction performance of the HGBR, ANN, LSTM, GRU, and LSTM + GRU models across increasing TOP-K ranked feature subsets on the MOVER dataset. The results show that the HGBR model consistently achieved the strongest performance in most TOP-K settings. As the number of ranked features increased from TOP-1 to TOP-7, the HGBR model exhibited a steady decrease in prediction error, with the mean absolute error (MAE) decreasing from 88.64 min at TOP-1 to 77.70 min at TOP-7, accompanied by corresponding improvements in the root mean squared error (RMSE) and the coefficient of determination (R²). This trend indicates that the proposed ranking strategy was effective in ranking the most informative features, and that the incremental addition of higher-ranking variables improved the predictive performance of the models.

Among the neural network-based models, the Artificial Neural Network (ANN) model achieved the best overall performance, with its lowest error at TOP-4, a mean absolute error (MAE) of 81.20, a root mean square error (RMSE) of 120.65, and a coefficient of determination (R²) of 0.2287. However, its performance did not stabilize when additional ranked features were added, indicating that the ANN model benefited from a limited subset of informative inputs but was less robust than the HGBR model in handling the full ranked feature progression. Similarly, the GRU model performed best at TOP-6, while the LSTM + GRU hybrid model performed best at TOP-7. Despite these improvements, both models remained inferior to the HGBR model in terms of overall prediction accuracy. The LSTM model showed the weakest overall performance among the models, recording relatively high prediction errors and low or even negative R² values in many TOP-K settings. In contrast, the superior performance of the HGBR model confirms that tree-based boosting methods are more suitable for this type of structured perioperative data, where nonlinear interactions can be effectively observed without the need for sequential time modeling.

The results show that the HGBR model is best suited for predicting surgical duration on the MOVER dataset within the evaluated TOP-K subsets. Although the best numerical performance of the HGBR algorithm was achieved at TOP-12, the improvement beyond TOP-7 was minimal. Therefore, TOP-7 was selected as the preferred compact predictive subset, as it provides a good balance between prediction accuracy and input complexity. These results support the effectiveness of the proposed feature ranking mechanism and the suitability of the HGBR model as the final prediction model within the ASTP framework.

**Table 12:** Performance of HGBR, ANN, LSTM, GRU, and LSTM + GRU across incremental TOP-K ranked features on the MOVER dataset.

TOP K	Features	Models	MAE	RMSE	$\:{\boldsymbol{R}}^{2}$
1	• patient_class_nm	HGBR	88.6394	129.6626	0.1091
		ANN	91.1035	129.4899	0.1116
		LSTM	94.4649	138.2569	−0.0128
		GRU	89.1931	128.5813	0.1240
		LSTM + GRU	96.8914	140.4625	−0.0454
2	• patient_class_nm • procedure_grp	HGBR	82.3449	123.9363	0.1861
		ANN	101.0607	139.3742	−0.0293
		LSTM	132.6749	187.6489	−0.8657
		GRU	97.4512	134.3202	0.0440
		LSTM + GRU	99.6188	148.6485	−0.1708
3	• patient_class_nm • procedure_grp • scheduled_hour	HGBR	80.9852	120.4569	0.2311
		ANN	84.8544	122.4702	0.2053
		LSTM	88.9653	131.9317	0.0777
		GRU	101.2007	133.6388	0.0537
		LSTM + GRU	97.3965	138.9577	−0.0231
4	• patient_class_nm • procedure_grp • scheduled_hour • primary_anes_type_nm	HGBR	79.1422	118.6212	0.2544
		ANN	81.1956	120.6543	0.2287
		LSTM	87.8574	128.1267	0.1302
		GRU	104.0602	148.3012	−0.1653
		LSTM + GRU	95.9061	139.3428	−0.0288
5	• patient_class_nm • procedure_grp • scheduled_hour • primary_anes_type_nm • dx_grp	HGBR	78.3015	117.2878	0.2711
		ANN	83.5013	121.8575	0.2132
		LSTM	97.6447	140.6176	−0.0477
		GRU	100.6640	148.9141	−0.1750
		LSTM + GRU	96.4669	137.7053	−0.0048
6	• patient_class_nm • procedure_grp • scheduled_hour • primary_anes_type_nm • dx_grp • admit_to_or_hours	HGBR	78.0705	116.8489	0.2765
		ANN	83.4154	124.0122	0.1851
		LSTM	97.3263	138.1570	−0.0114
		GRU	83.2352	122.0648	0.2105
		LSTM + GRU	97.5045	135.7505	0.0236
7	• patient_class_nm • procedure_grp • scheduled_hour • primary_anes_type_nm • dx_grp • admit_to_or_hours • asa_rating	HGBR	77.6978	116.4199	0.2818
		ANN	113.5750	158.3019	−0.3278
		LSTM	99.5784	138.7267	−0.0197
		GRU	102.6233	149.4705	−0.1838
		LSTM + GRU	85.5584	125.6926	0.1629
8	• patient_class_nm • procedure_grp • scheduled_hour • primary_anes_type_nm • dx_grp • admit_to_or_hours • asa_rating • sex	HGBR	77.8442	116.6278	0.2792
		ANN	100.0639	142.8832	−0.0817
		LSTM	97.9034	135.8580	0.0220
		GRU	100.7540	149.4755	−0.1839
		LSTM + GRU	87.4052	127.8033	0.1345
9	• patient_class_nm • procedure_grp • scheduled_hour • primary_anes_type_nm • dx_grp • admit_to_or_hours • asa_rating • sex • weight_kg	HGBR	77.5433	116.2356	0.2841
		ANN	107.0367	147.8749	−0.1586
		LSTM	84.5542	121.9832	0.2116
		GRU	83.1445	123.8396	0.1874
		LSTM + GRU	99.8743	145.8175	−0.1266
10	• patient_class_nm • procedure_grp • scheduled_hour • primary_anes_type_nm • dx_grp • admit_to_or_hours • asa_rating • sex • weight_kg • height_cm	HGBR	77.5898	116.3306	0.2829
		ANN	82.2144	122.2658	0.2079
		LSTM	108.3511	145.4315	−0.1207
		GRU	106.9849	144.5006	−0.1064
		LSTM + GRU	96.9443	142.6126	−0.0776
11	• patient_class_nm • procedure_grp • scheduled_hour • primary_anes_type_nm • dx_grp • admit_to_or_hours • asa_rating • sex • weight_kg • height_cm • bmi	HGBR	77.6703	116.5124	0.2807
		ANN	83.9228	123.0771	0.1974
		LSTM	83.0945	122.7197	0.2020
		GRU	82.3340	121.1368	0.2225
		LSTM + GRU	90.1567	135.0481	0.0336
12	• patient_class_nm • procedure_grp • scheduled_hour • primary_anes_type_nm • dx_grp • admit_to_or_hours • asa_rating • sex • weight_kg • height_cm • bmi • surgery_month	HGBR	77.4762	116.1977	0.2845
		ANN	107.5550	152.4368	−0.2312
		LSTM	83.7737	122.4975	0.2049
		GRU	82.3653	121.3487	0.2198
		LSTM + GRU	98.9827	145.1081	−0.1157
13	• patient_class_nm • procedure_grp • scheduled_hour • primary_anes_type_nm • dx_grp • admit_to_or_hours • asa_rating • sex • weight_kg • height_cm • bmi • surgery_month • surgery_weekday	HGBR	77.5425	116.2571	0.2838
		ANN	116.6301	157.0896	−0.3075
		LSTM	98.5451	135.2787	0.0303
		GRU	84.0753	122.4884	0.2050
		LSTM + GRU	96.8690	139.6980	−0.0340

**Fig. 13:**
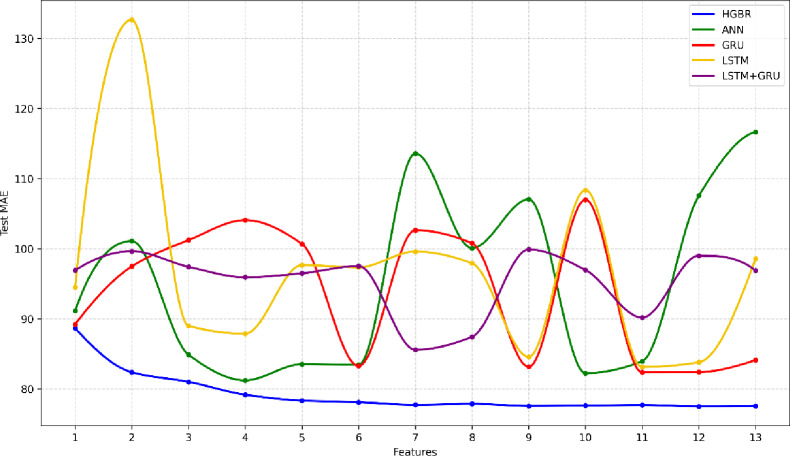
MAE performance of different models (HGBR, ANN, GRU, LSTM, and LSTM + GRU) across incremental feature subsets (TOP K) on MOVER dataset.

Figure 13 illustrates the mean absolute error (MAE) performance of the HGBR, ANN, LSTM, GRU, and LSTM + GRU models across increasing TOP-K ranked feature subsets on the MOVER dataset. The results show that the HGBR model achieved the most stable behavior and the lowest overall prediction error, while the other models exhibited greater variability as additional features were included.

To achieve a balanced comparison between predictive performance and feature utilization efficiency, Table 13 presents the best numerical result and best subset for each evaluated model on the MOVER dataset, in terms of mean absolute error (MAE), root mean square error (RMSE), and coefficient of determination (R²). This presentation highlights not only the absolute best performance achieved by each model but also the most practically efficient operating point in terms of reducing input dimensions. In particular, this allows for a more accurate interpretation of the HGBR-based ASTP framework by distinguishing between its best numerical configuration and its best subset, where only minor differences in accuracy are observed despite the reduced number of selected features.

**Table 13:** Best numerical and best subset performance results of the evaluated models on the MOVER dataset.

Model	Selection	Number of Features	MAE	RMSE	*R*²
HGBR	Best numerical	12	77.4762	116.1977	0.2845
	Best subset	7	77.6978	116.4199	0.2818
ANN	Best numerical	4	81.1956	120.6543	0.2287
	Best subset	4	81.1956	120.6543	0.2287
LSTM	Best numerical	11	83.0945	122.7197	0.2020
	Best subset	9	84.5542	121.9832	0.2116
GRU	Best numerical	11	82.3340	121.1368	0.2225
	Best subset	6	83.2352	122.0648	0.2105
LSTM + GRU	Best numerical	7	85.5584	125.6926	0.1629
	Best subset	7	85.5584	125.6926	0.1629

### Comparison with previous studies

Table 14compares the proposed HGBR-based ASTP model with other methods published in previous articles.The proposed HGBR model achieves a mean absolute error (MAE) of 8.89 min, a root mean square error (RMSE) of 19.5 min, and a coefficient of determination (R²) of 0.26 using only four features, demonstrating good predictive performance with reduced input dimensions. Although XGBoost and the basic gradient boosted-tree method achieve better numerical results, they rely on six features and do not provide the same efficiency as ASTP in reducing the number of features. Similarly, RandomForest (General) and RandomForest (Dept-spec) both use six features and offer competitive results, although their predictive errors remain slightly higher than those of XGBoost. These results indicate that the proposed HGBR model provides a competitive and practically efficient solution for predicting surgical time on structured tabular data. In addition to direct model comparisons, a methodological comparison was conducted with similar recent studies published in 2025. Kwong et al^13^. reported strong performance based on artificial neural networks in predicting the duration of common elective general surgical procedures at three academic centers, while Ramamurthy et al^17^. demonstrated the importance of integrating machine learning with clinical text representations and later extended this approach using large-scale linguistic models to predict surgical duration. Levin et al^26^. also confirmed the usefulness of boosted tree models in predicting the duration of surgical procedures in each specialty.

**Table 14:** Best performance results for each model in terms of MAE, RMSE, and R² vs. other implemented techniques.

	feature	MAE	RMSE	$\:{R}^{2}$
HGBR	4	8.89	19.5	0.26
XGBoost^18^	6	8.7	18.4	0.31
RandomForest (General)^19^	6	9.1	18.8	0.29
RandomForest (Dept-spec)^19^	6	9.0	18.7	0.30
gradient-boosted tree baseline^26^	6	8.5	17.35	0.70

**Fig. 14:**
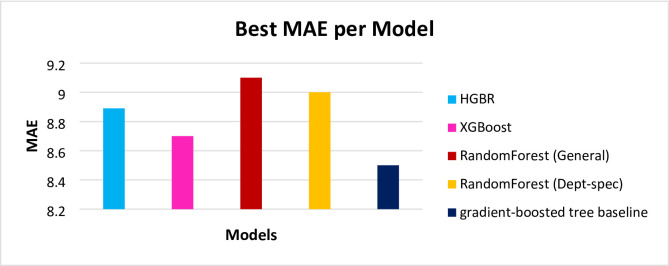
Best MAE per model with the corresponding number of features (K) of HGBR vs. other implemented techniques.

**Fig. 15:**
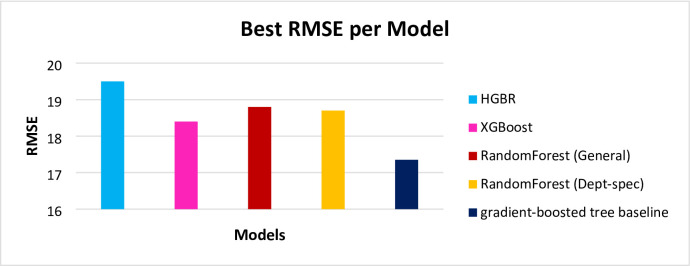
Best RMSE per model with the corresponding number of features (K) of HGBR vs. other implemented techniques.

**Fig. 16:**
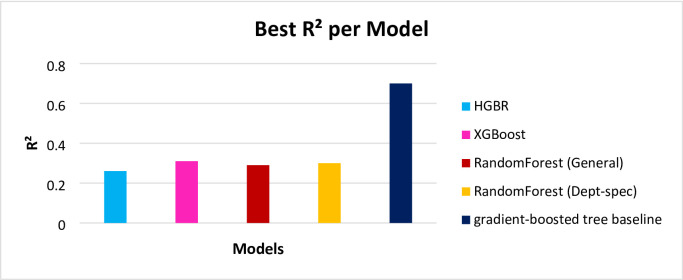
Best R² per model with the corresponding number of features (K) of HGBR vs. other implemented techniques.

Compared to these recent studies, the proposed ASTP framework is distinguished by its integration of interpretable feature ranking, feature reduction using TOP-K, and efficient HGBR-based prediction using structured and compressed tabular inputs. Therefore, the current framework not only offers competitive predictive performance but also improved feature economy and practical interpretability. Figures 14 and 15, and 16 show a comparison between each metric.

Table 15 illustrates an in-depth comparison of the evaluated models. The HGBR system demonstrates strong overall performance with a mean absolute error (MAE) of 8.89 min, a root mean squared error (RMSE) of 19.5 min, and R² of 0.2*6*, using only four features, making it efficient and low-cost for clinical use. Artificial neural networks (ANNs) achieve the highest R² (0.32) with six features, indicating improved correlation, but at the expense of hyperparameter tuning sensitivity. Both the LSTM-GRU, as well as hybrid LSTM + GRU system, show lower efficiency on surgical data (mean absolute error ≈ 11.7–12.0 min) and require long training times (20–85 s), limiting real-time predictability. With a mean absolute error (MAE) of 8.70 min and a root mean square error (RMSE) of 18.4 min, XGBoost provides somewhat higher accuracy than HGBR; nevertheless, it necessitates the use of all six characteristics and meticulous parameter adjustment. While RandomForest (Dept-spec) offers personalized predictions but necessitates big datasets, RandomForest (General) produces competitive results (MAE = 9.0 min, RMSE ≈ 18.7 min) with extremely quick prediction times, making it appropriate for real-world applications.

**Table 15:** An in-depth performance comparison of HGBR and other machine learning models (ANN, LSTM, GRU, Hybrid, XGBoost, and RandomForest) using key evaluation metrics.

Method	Features	MAE (min)	RMSE (min)	*R*²	Computational time	Best use case	Limitations
**HGBR**	4	8.89	19.5	0.26	7.5s	Balanced accuracy and efficiency with fewer features	Needs parameter tuning
**ANN**	6	9.99	18.7	0.32	2.2s	Works well with full features	Sensitive to hyperparameters
**LSTM**	2	11.90	20.1	0.22	22s	Nonlinear feature interaction modeling	Weaker on static data
**GRU**	2	11.7373	19.8910	0.2318	20s	Lightweight gated nonlinear modeling	Limited gain on input
**Hybrid (LSTM + GRU)**	2	12.02	20.1	0.21	85s	Complex gated nonlinear modeling	High computational cost
XGBoost^18^	6	8.7	18.4	0.31	10s	Strong baseline with boosting	Sensitive to imbalance
RandomForest (General)^19^	6	9.1	18.8	0.29	5s	Robust, interpretable	Needs many trees for accuracy
RandomForest (Dept-spec)^19^	6	9.0	18.7	0.30	5s	Tailored to sub-groups	Requires enough data
gradient-boosted tree baseline^26^	6	8.5	17.35	0.70	11s	Specialty-specific arthroplasty duration prediction	Reports case-prediction accuracy rather than regression-error metrics

## Conclusion

This study presented an accurate surgical time prediction (ASTP) strategy for accurate estimation of surgical duration. The proposed framework was designed to address prediction accuracy, feature interpretability, learning using reduced features, and computational efficiency in estimating surgical time. Using a real operating room (OR) dataset from Nile Hospital, The Medical Informatics Operating Room Vitals and Events Repository (MOVER), our method consists of two stages. The first stage involves preprocessing using artificial intelligence techniques (SHAP-based LSTM interpretability and Random Forest importance switching) to identify the most important features. The second stage involves evaluating the incremental TOP-K using multiple methods (ANN, LSTM, GRU, hybrid LSTM + GRU, and HGBR). On the Nile Hospital dataset, HGBR achieved the best balance between accuracy and efficiency, achieving an MAE = 8.89 min, RMSE = 19.5 min, and R² = 0.26 with only four features. Statistical significance analysis confirmed that its improvement compared to other models was not due to random variation. Furthermore, experiments on the MOVER dataset demonstrated the generalizability of the proposed framework, with HGBR also achieving the best numerical the top 12 subsets and the best subset among the top 7 subsets. The results highlight the importance of interpretability and feature availability in predictive healthcare systems. The proposed ASTP method improves prediction accuracy, which improves operating room scheduling, reduces resource waste, and ensures better patient care. Future work will focus on validating the ASTP framework, incorporating more comprehensive preoperative and intraoperative variables, and enhancing integration with future operating room scheduling and decision-support systems. Additional features, such as procedure type, anesthesia duration, intraoperative events, and textual clinical notes, may contribute to improve surgical duration modeling. Further evaluation in other hospitals and specialties, along with advanced models such as modular learning, transfer learning, and reinforcement learning-based scheduling, will be explored to improve adaptability to heterogeneous environments.

## Data Availability

Link to the dataset: h t t p s : / / w w w . k a g g l e . c o m / d a t a s e t s / r a n a e l b a l k a / o p e r a t i n g - r o o m - s u r g e r y - d a t a s e t / d a t a.

## References

[1] Hinterwimmer, F. et al. Prediction of complications and surgery duration in primary TKA with high accuracy using machine learning with arthroplasty-specific data. *Knee Surg. Sports Traumatol. Arthrosc.* **31** (4), 1323–1333 (2023). 35394135 10.1007/s00167-022-06957-w PMC10050062

[2] Yeo, I. et al. Predicting surgical operative time in primary total knee arthroplasty utilizing machine learning models. *Arch. Orthop. Trauma Surg.* **143** (6), 3299–3307 (2023). 35994094 10.1007/s00402-022-04588-x

[3] Tully, J. L. et al. Machine learning prediction models to reduce length of stay at ambulatory surgery centers through case resequencing. *J. Med. Syst.* **47** (1), 71 (2023). 37428267 10.1007/s10916-023-01966-9 PMC10333394

[4] Toma, M. *Ong Chi Wei Predictive Model. Med. Encyclopedia* **3**.2 : 590–601. (2023).

[5] Pasquer, A. et al. Operating room organization and surgical performance: a systematic review. *Patient Saf. Surg.* **18** (1), 5 (2024). 38287316 10.1186/s13037-023-00388-3 PMC10826254

[6] Li, C. J. et al. Physician burnout and medical errors: exploring the relationship, cost, and solutions. *Am. J. Med. Qual.* **38** (4), 196–202 (2023). 37382306 10.1097/JMQ.0000000000000131

[7] Adegbesan, A. et al. From scalpels to algorithms: the risk of dependence on artificial intelligence in surgery. *J. Med. Surg. Public. Health*. **3**, 100140 (2024).

[8] Ala, A. & Goli, A. Incorporating machine learning and optimization techniques for assigning patients to operating rooms by considering fairness policies. *Eng. Appl. Artif. Intell.* **136**, 108980 (2024).

[9] Eshghali, M. et al. Machine learning based integrated scheduling and rescheduling for elective and emergency patients in the operating theatre. *Ann. Oper. Res.* **332** (1), 989–1012 (2024). 10.1007/s10479-023-05168-x PMC9851122 36694896

[10] Mathis, M. et al. Overview and clinical applications of artificial intelligence and machine learning in cardiac anesthesiology. *J. Cardiothorac. Vasc. Anesth.* **38** (5), 1211–1220 (2024). 38453558 10.1053/j.jvca.2024.02.004 PMC10999327

[11] El-Balka, R. et al. A dynamic operation room scheduling DORS strategy based on explainable AI and fuzzy interface engine. *Artif. Intell. Rev.* **58**, 365 (2025).

[12] Kostopoulos, S. et al. Prediction of remaining surgery duration based on machine learning methods and laparoscopic annotation data. *Biomedical Engineering/Biomedizinische Technik*. **70** (3), 229–239 (2025). 40116444 10.1515/bmt-2024-0431

[13] Kwong, M., Noorchenarboo, M., Grolinger, K., Hawel, J., Schlachta, C. M., & Elnahas, A. Optimizing surgical effi ciency:predicting case duration of common general surgery procedures using machine learning. *Surgical Endoscopy*, **39**,5227–5234 (2025). 10.1007/s00464-025-11885-0 40571798

[14] Riahi, V. et al. Improving preoperative prediction of surgery duration. *BMC Health Serv. Res.* **23** (1), 1343 (2023). 38042831 10.1186/s12913-023-10264-6 PMC10693694

[15] Gabriel, R. et al. An ensemble learning approach to improving prediction of case duration for spine surgery: algorithm development and validation. *JMIR Perioperative Med.* **6**, e39650 (2023). 10.2196/39650 PMC9912154 36701181

[16] Jiao, Y. et al. Continuous real-time prediction of surgical case duration using a modular artificial neural network. *Br. J. Anaesth.* **128**, 829–837 (2022). 35090725 10.1016/j.bja.2021.12.039 PMC9074795

[17] Ramamurthi, A. et al. Development and validation of an artificial intelligence system for surgical case length prediction. *Surgery* 179 : 108942. (2025). 10.1016/j.surg.2024.09.051 39613655

[18] Langenberger, B. et al. Leveraging machine learning for duration of surgery prediction in knee and hip arthroplasty–a development and validation study. *BMC Med. Inf. Decis. Mak.* **25** (1), 106 (2025). 10.1186/s12911-025-02927-7 PMC11877953 40033378

[19] Park, J. B. et al. Development of Predictive Model of Surgical Case Durations Using Machine Learning Approach. *J. Med. Syst.* **49** (1), 8 (2025). 39808376 10.1007/s10916-025-02141-y PMC11732958

[20] Seger, C. An investigation of categorical variable encoding techniques in machine learning:binary versus one-hot and feature hashing. Master’s thesis, *KTH Royal Institute of Technology* (2018).

[21] Al-Selwi, S. et al. RNN-LSTM: From applications to modeling techniques and beyond—Systematic review. *J. King Saud University-Computer Inform. Sci.* **36**, 102068 (2024).

[22] Makumbura, R. K. et al. Advancing water quality assessment and prediction using machine learning models, coupled with explainable artificial intelligence (XAI) techniques like shapley additive explanations (SHAP) for interpreting the black-box nature. *Results Eng.* **23**, 102831 (2024).

[23] Iranzad, R., & Liu, X. A review of random forest-based feature selection methods for data science education andapplications. *International Journal of Data Science and Analytics*, **20**, 197–211 (2025).

[24] Theerthagiri, P. Liver disease classification using histogram-based gradient boosting classification tree with feature selection algorithm. *Biomed. Signal Process. Control*. **100**, 107102 (2025).

[25] Link to the dataset. https://www.kaggle.com/datasets/ranaelbalka/operating-room-surgery-dataset/data

[26] Levin, J. M. et al. A machine learning prediction model for total shoulder arthroplasty procedure duration: an evaluation of surgeon, patient, and shoulder-specific factors. *J. Shoulder Elbow Surg.* **34**, 1792–1800 (2025). 39716610 10.1016/j.jse.2024.10.028

[27] Ramamurthi, A. et al. Applying large language models for surgical case length prediction. *JAMA Surg.* **160** (8), 894–902 (2025). 40632526 10.1001/jamasurg.2025.2154 PMC12242817

[28] Medical Informatics Operating Room. Vitals and Events Repository (MOVER), dataset access DOI: 10.24432/C5VS5G. 10.1093/jamiaopen/ooad084 PMC10582520 37860605

